# Supplementary material for: PD-L1 lncRNA splice isoform promotes lung adenocarcinoma progression via enhancing c-Myc activity
Source: Genome Biol. 2021 Apr 13;22:104. doi: 10.1186/s13059-021-02331-0 (PMC8042710; doi:10.1186/s13059-021-02331-0)
Supplement: Supplementary file 1 — Additional file 1: Table S1. Clinic characteristics of lung adenocarcinoma patients. Table S2. List of primers for qRT-PCR. Table S3. List of target sequences of various siRNAs. [file 13059_2021_2331_MOESM1_ESM.pdf]

Table S1. Clinic characteristics of lung adenocarcinoma patients.

	PD-L1 Negative	PD-L1 Positive	P value
**Parameter**			
**All patients**	N=212 (77.1%)	N=63 (22.9%)	
**Age (years)**			**0.827**
<60	84 (77.8%)	24 (22.2%)	
≧60	128 (76.6%)	39 (23.4%)	
**Gender**			
Female	136 (85%)	24 (15%)	**0.000**
Male	76 (66%)	39 (34%)	
**Pathologic stage**			
I	164 (82.4%)	35 (17.6%)	**0.001**
II-III	48 (70.6%)	28 (29.4%)	
**Pathologic T stage**			
T1	142 (82.6%)	30 (17.4%)	**0.005**
T2-T4	70 (68.0%)	33 (32.0%)	
**Pathologic N stage**			
N0	171 (82.2%)	37 (17.8%)	**0.000**
N1-N3	41 (62.0%)	26 (38.0%)	

Table S2. List of primers for qRT-PCR.

Gene name	Primers	
	Forward	Reverse
**GAPDH**	TGAACGGGAAGCTCACTGG	TCCACCACCCTGTTGCTGTA
**PD-L1 mRNA primer 1**	AGGCCGAAGTCATCTGGAC	CTGGGATGACCAATTCAGCT
**PD-L1 mRNA/lnc primer 2**	TCACGGTTCCCAAGGACCTA	CCCCGATGAACCCCTAAACC
**PD-L1 mRNA primer 3**	CCAGTCACCTCTGAACATG	TCAGTGTGCTGGTCACATTG
**PD-L1-lnc primer 4**	CTGAGTGGAGATTAGATCCTG	CATCATTCTCCCAAGTGAGTC
**SAA2**	GCTTCTTTTCGTTCCTTGGCG	GCCGATGTAATTGGCTTCTCTCA
**OAS2**	ACGTGACATCCTCGATAAAACTG	GAACCCATCAAGGGACTTCTG
**IFI44L**	ACAGAGCCAAATGATTCCCTATG	TCGATAAACGACACACCAGTTG
**SAA4**	GGCAGAGCCTATTGGGACATA	GCTGATGAGTTTAGCAGCCC
**IFI44**	ATGGCAGTGACAACTCGTTTG	TCCTGGTAACTCTCTTCTGCATA
**OASL**	CTGATGCAGGAACTGTATAGCAC	CACAGCGTCTAGCACCTCTT
**IFITM1**	CCAAGGTCCACCGTGATTAAC	ACCAGTTCAAGAAGAGGGTGTT
**MX2**	CAGAGGCAGCAGACGATCAAC	TTGGTCAGGATACCGATGGTC
**MX1**	GTTTCCGAAGTGGACATCGCA	CTGCACAGGTTGTTCTCAGC
**COL8A1**	GCTGCCACCTCAAATTCCTC	CTTTCTTGGGTACGGCTTCCT
**IFIT1**	AGAAGCAGGCAATCACAGAAAA	CTGAAACCGACCATAGTGGAAAT
**IFIT3**	AAAAGCCCAACAACCCAGAAT	CGTATTGGTTATCAGGACTCAGC
**SOD2**	GCTCCGGTTTTGGGGTATCTG	GCGTTGATGTGAGGTTCCAG
**IFITM3**	ACTGTCCAAACCTTCTTCTCTCC	TCGCCAACCATCTTCCTGTC
**IFI6**	GGTCTGCGATCCTGAATGGG	TCACTATCGAGATACTTGTGGGT
**HLA-A**	GACGCCCCCAAAACGCATA	TGGGCAAACCCTCATGCTG
**GFP mRNA**	AAGGACGACGGCAACTACAA	CGATGTTGTGGCGGATCTTG
**MSI1**	GGGACTCAGTTGGCAGACTAC	CTGGTCCATGAAAGTGACGAA
**ESRP2**	TTGCAGCAAGGCTGATGTG	GTTGAGGCAGAGTGCTACACC
**DAZAP1**	AGAAGTTCGGAGTGGTCACG	ACTGATTGTTCGTCCTCGAAAG

Table S3. List of target sequences of various siRNAs.

Gene name	Target sequence 5’-3’
**PD-L1-lnc shRNA**	GACTCACTTGGGAGAATGATGGA
**c-Myc-si-1**	GAGGAGACATGGTGAACCA
**c-Myc-si-2**	GGGTCAAGTTGGACAGTGT
**c-Myc-si-3**	CGACGAGACCTTCATCAAA
**DAZAP-si-1**	CGGAGGTAGTCATGATCTA
**DAZAP-si-2**	CAACCAGTCTCGAGGCTTT
