# Supplementary material for: PD-L1 lncRNA splice isoform promotes lung adenocarcinoma progression via enhancing c-Myc activity
Source: Genome Biol. 2021 Apr 13;22:104. doi: 10.1186/s13059-021-02331-0 (PMC8042710; doi:10.1186/s13059-021-02331-0)
Supplement: Supplementary file 2 — Additional file 2: Figure S1. The PD-L1 protein level in human lung cancer tissue. Figure S2. The Sanger sequencing result of the PD-L1 mRNA (878 bp band) and PD-L1 lncRNA (705 bp band) obtained by RT-PCR. Figure S3. The Sanger sequencing result of the PD-L1 mRNA obtained by qRT-PCR with specific primer for amplification of PD-L1 mRNA (157 bp band) and PD-L1 lncRNA (106 bp band) obtained by RT-PCR. Figure S4. Expression of PD-L1-lnc in various cancers. Figure S5. PD-L1-lnc is a long non-coding RNA fragment. Figure S6. Induction of PD-L1 by IFNγ in lung adenocarcinoma cells. Figure S7. PD-L1-lnc has no effect on PD-L1 mRNA and protein expression. Figure S8. Expression level of genes within the c-Myc signaling downstream in lung cancer xenograft. Figure S9. PD-L1-lnc binds to c-Myc. Figure S10. Graphical representation images of the binding interface of the docking models of PD-L1-lnc with c-Myc by NPDock. Figure S11. The expression of PD-L1 lncRNA and mRNA in A549 cells transfected with the mutant vector and wild type vector. Figure S12. Cell fraction assay. Figure S13. Binding of PD-L1-lnc to c-Myc enhances the association of c-Myc with MAX and promotes the entry of c-Myc into nucleus. Figure S14. The influence of c-Myc siRNA on gene expression in lung cancer A549 and PC9 cells. Figure S15. The effect of DAZAP1 on the generation of PD-L1-lnc. [file 13059_2021_2331_MOESM2_ESM.pdf]

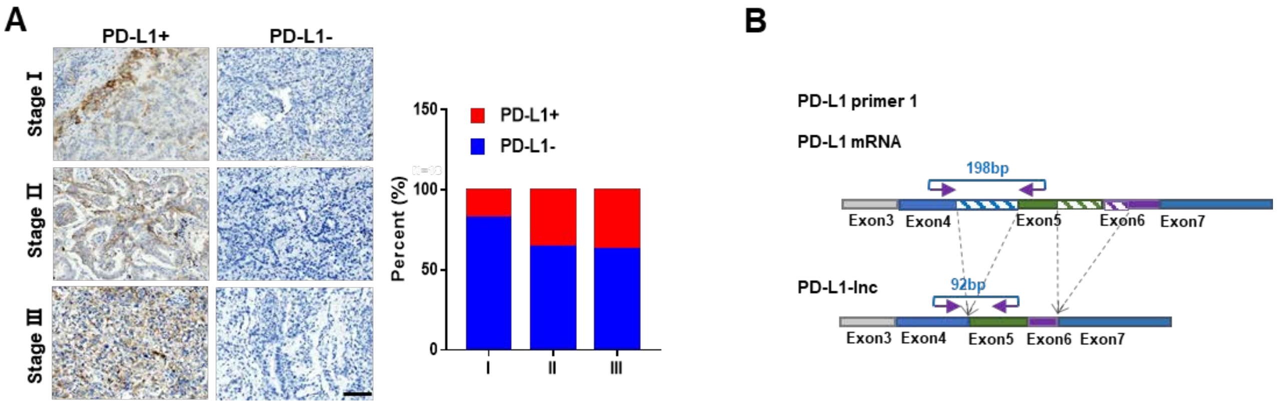


**Figure S1. The PD-L1 protein level in human lung cancer tissue. (A)** Immunohistochemical staining for PD-L1 in human lung cancer tissue sections. Left: representative image; Right: quantitative analysis. Tumors were classified into three stages (Stage Ⅰ, Ⅱ and Ⅲ) according to the 7th edition of the American Joint Committee on Cancer Staging Manual 17. Scale bar, 100μm. **(B)** The schematic of primer for amplification of PD-L1 mRNA.

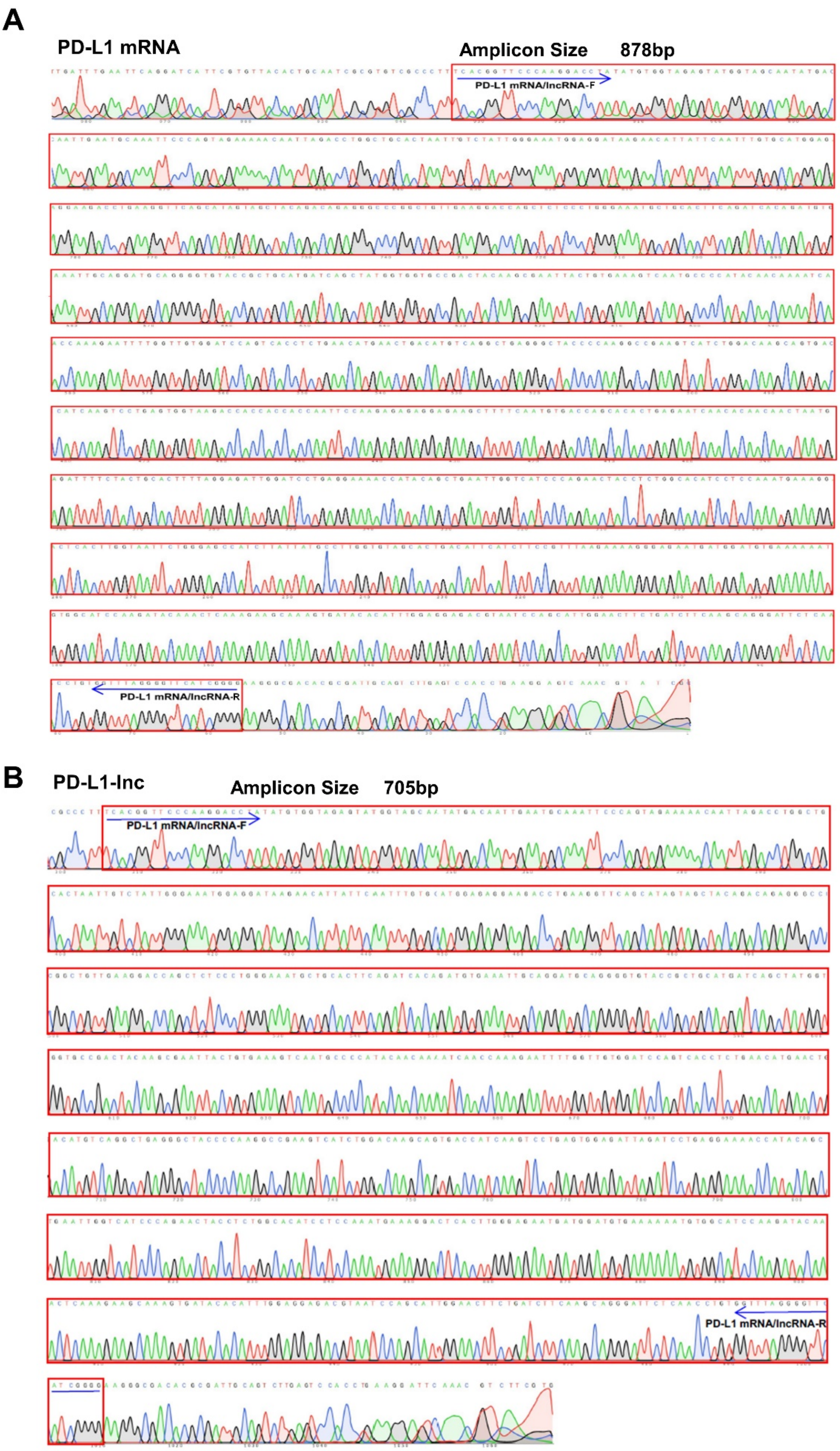


**Figure S2.** The Sanger sequencing results of the (A) PD-L1 mRNA (878*bp* band) and (B) PD-L1 lncRNA (705*bp* band) obtained by RT-PCR.

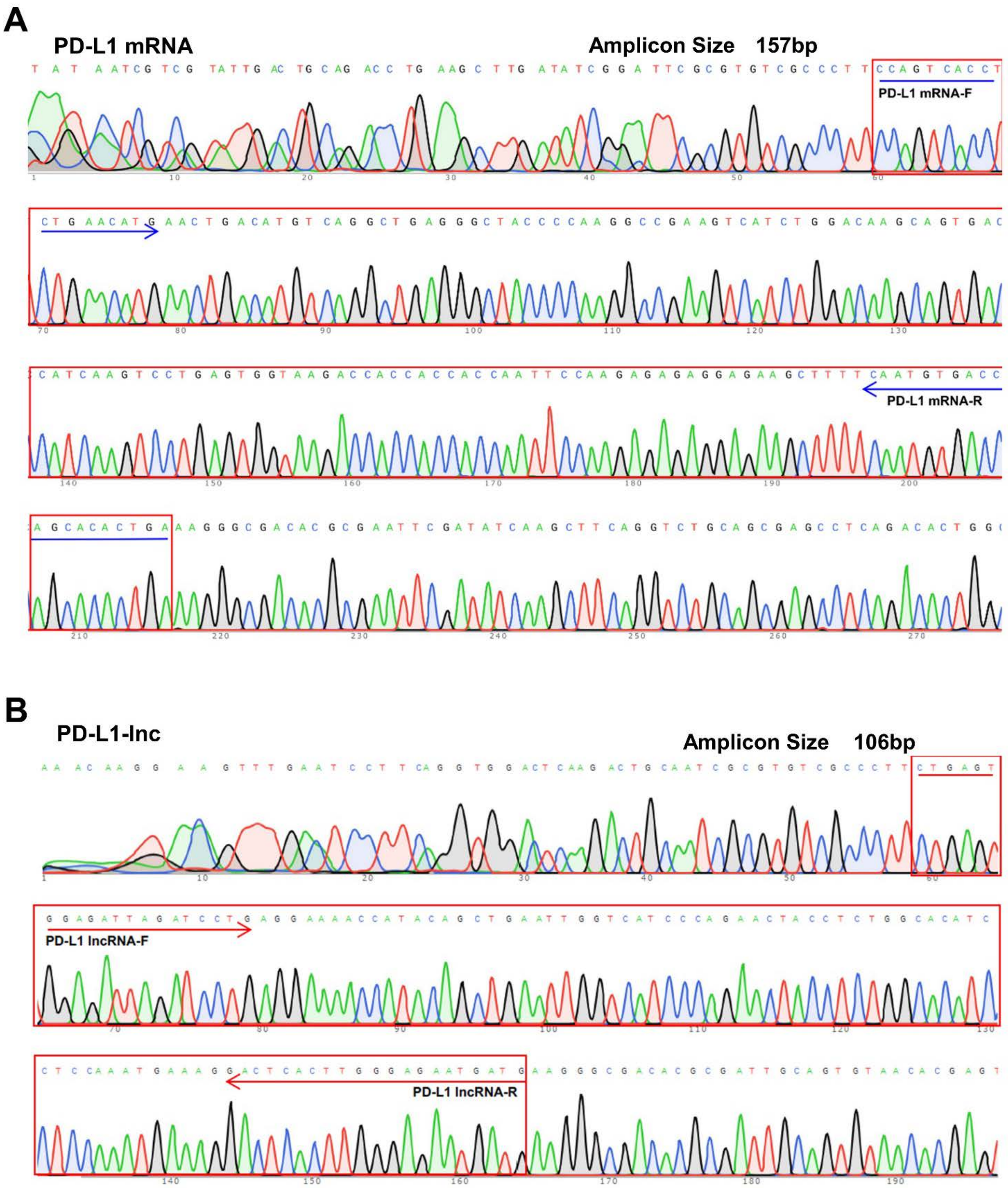


**Figure S3.** The Sanger sequencing results of (A) PD-L1 mRNA (157*bp* band) and (B) PD-L1 lncRNA (106*bp* band) obtained by qRT-PCR with specific primers.

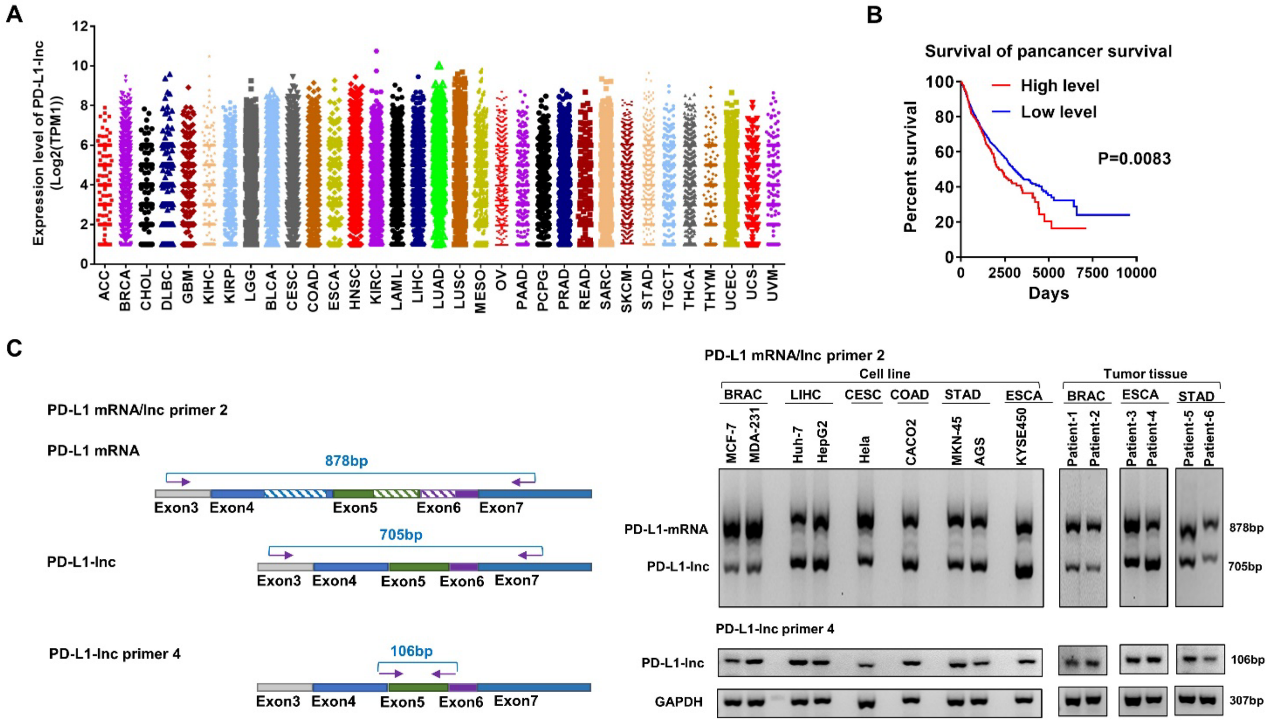


**Figure S4. Expression of PD-L1-lnc in various cancers. (A)** Pan-cancer analysis of PD-L1-lnc expression in TCGA database. **(B)** PD-L1-lnc as a predictor of cancer survival in TCGA database. **(C)** Left, the schematic of primer for amplification of PD-L1 mRNA and PD-L1-lnc; Right, the agarose gel of the PD-L1 mRNA and PD-L1-lnc in BRAC, LIHC, CESC, COAD, STAD and ESCA cancer cell lines and BRAC, ESCA and STAD cancer tissues by RT-PCR with specific primers. **ACC**: Adrenocortical carcinoma; **BRCA**: Breast invasive carcinoma; **CHOL**: Cholangiocarcinoma; **DLBC**: Lymphoid neoplasm diffuse large B-cell lymphoma; **GBM**: Glioblastoma multiforme; **KICH:** Kidney chromophobe; **KIRP**: Kidney renal papillary cell carcinoma; **LGG**: Brain lower grade glioma; **BLCA**: Bladder urothelial carcinoma; **CESC**: Cervical squamous cell carcinoma and endocervical adenocarcinoma; **COAD**: Colon adenocarcinoma; **ESCA**: Esophageal carcinoma; **HNSC**: Head and neck squamous cell carcinoma; **KIRC**: Kidney renal clear cell carcinoma; **LAML**: Acute myeloid leukemia; **LIHC:** Liver hepatocellular carcinoma; **LUAD**: Lung adenocarcinoma; **LUSC**: Lung squamous cell carcinoma; **MESO**: Mesothelioma; **OV**: Ovarian serous cystadenocarcinoma; **PAAD**: Pancreatic adenocarcinoma; **PCPG**: Pheochromocytoma and paraganglioma; **PRAD**: Prostate adenocarcinoma; **READ**: Rectum adenocarcinoma; **SARC**: Sarcoma; **SKCM**: Skin cutaneous melanoma; **STAD**: Stomach adenocarcinoma; **TGCT**: Testicular germ gell tumors; **THCA**: Thyroid carcinoma; **THYM:** Thymoma; **UCEC:** Uterine corpus endometrial carcinoma; **UCS**: Uterine carcinosarcoma; **UVM**: Uveal Melanoma.

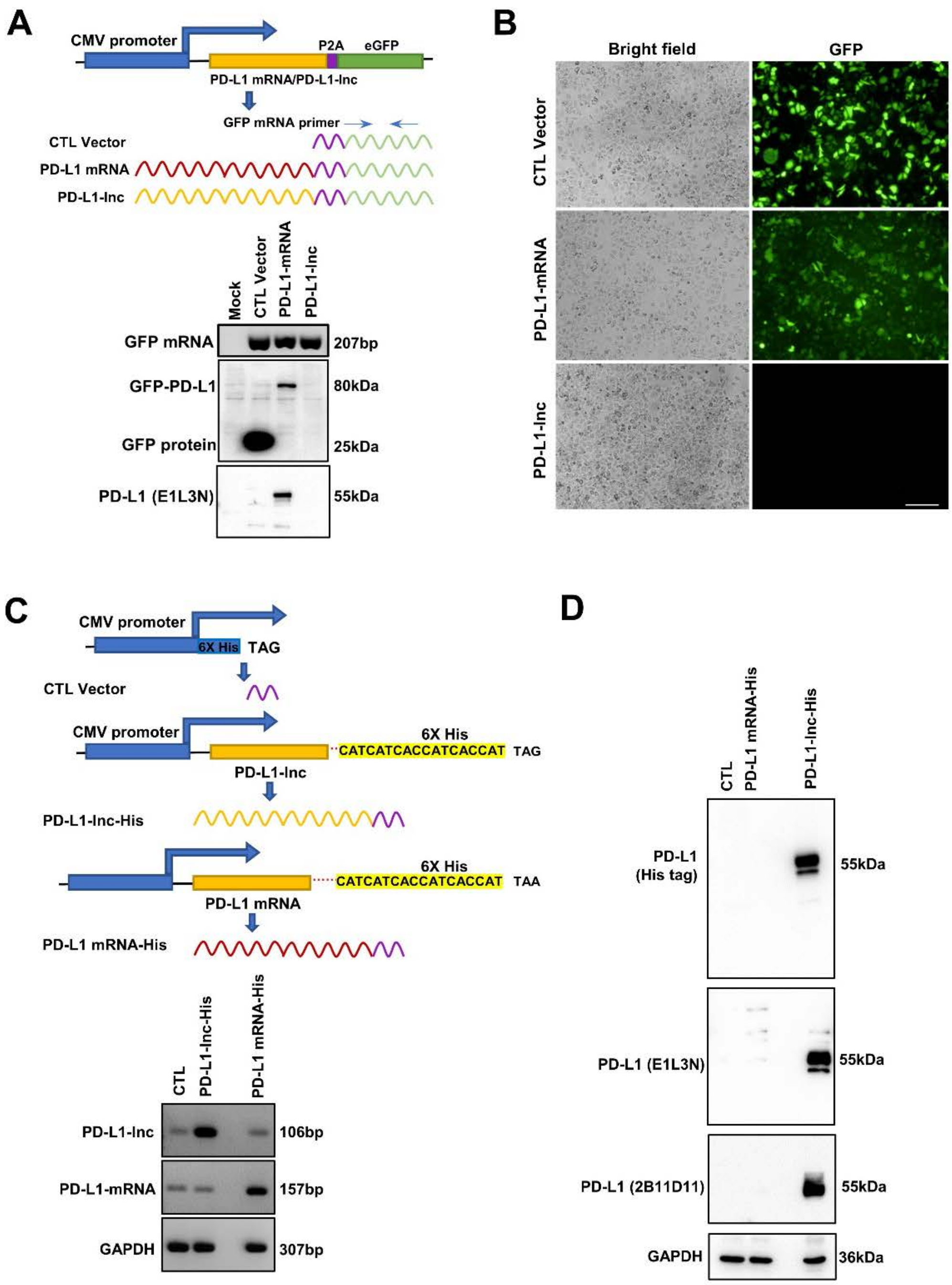


**Figure S5. PD-L1-lnc is a long non-coding RNA fragment. (A)** Linking PD-L1-lnc or PD-L1-mRNA with GFP mRNA to generate the ‘recombinant’ GFP expression vector. **(B)** GFP expression detected by fluorescence microscopy in A549 cells that were transfected with GFP expression or ‘recombinant’ GFP expression vectors. Scale bar, 200μm. **(C)** Preparation of vectors expressing His-tagged recombinant proteins. **(D)** His-tag and PD-L1 protein expression detected by Western blotting in A549 cells. PD-L1-lnc overexpression vector: PD-L1-lnc; PD-L1 overexpression vector: PD-L1-mRNA; control vector: CTL Vector.

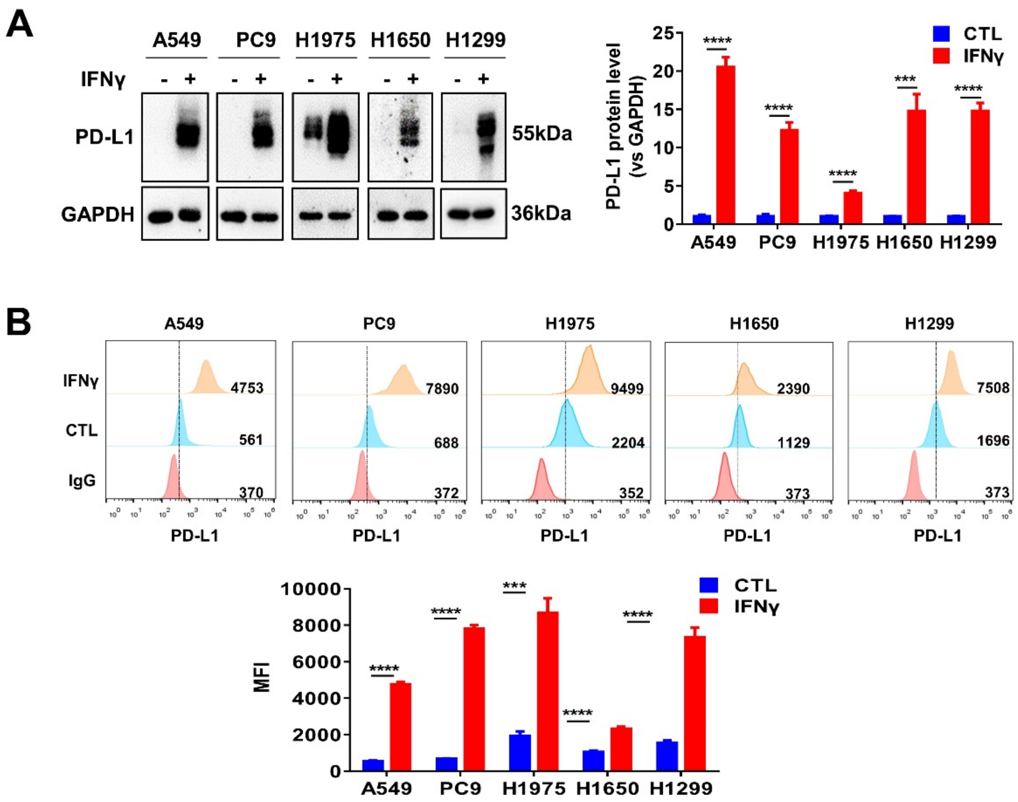


**Figure S6. Induction of PD-L1 by IFNγ in lung adenocarcinoma cells. (A)** The expression level of PD-L1 protein in lung adenocarcinoma cells with or without IFNγ stimulation determined by Western blotting. **(B)** The expression level of PD-L1 protein in lung adenocarcinoma cells with or without IFNγ stimulation determined by flow cytometry. In A-B, left: representative image; right: quantitative analysis. ***$P < 0.001$; ****$P < 0.0001$.

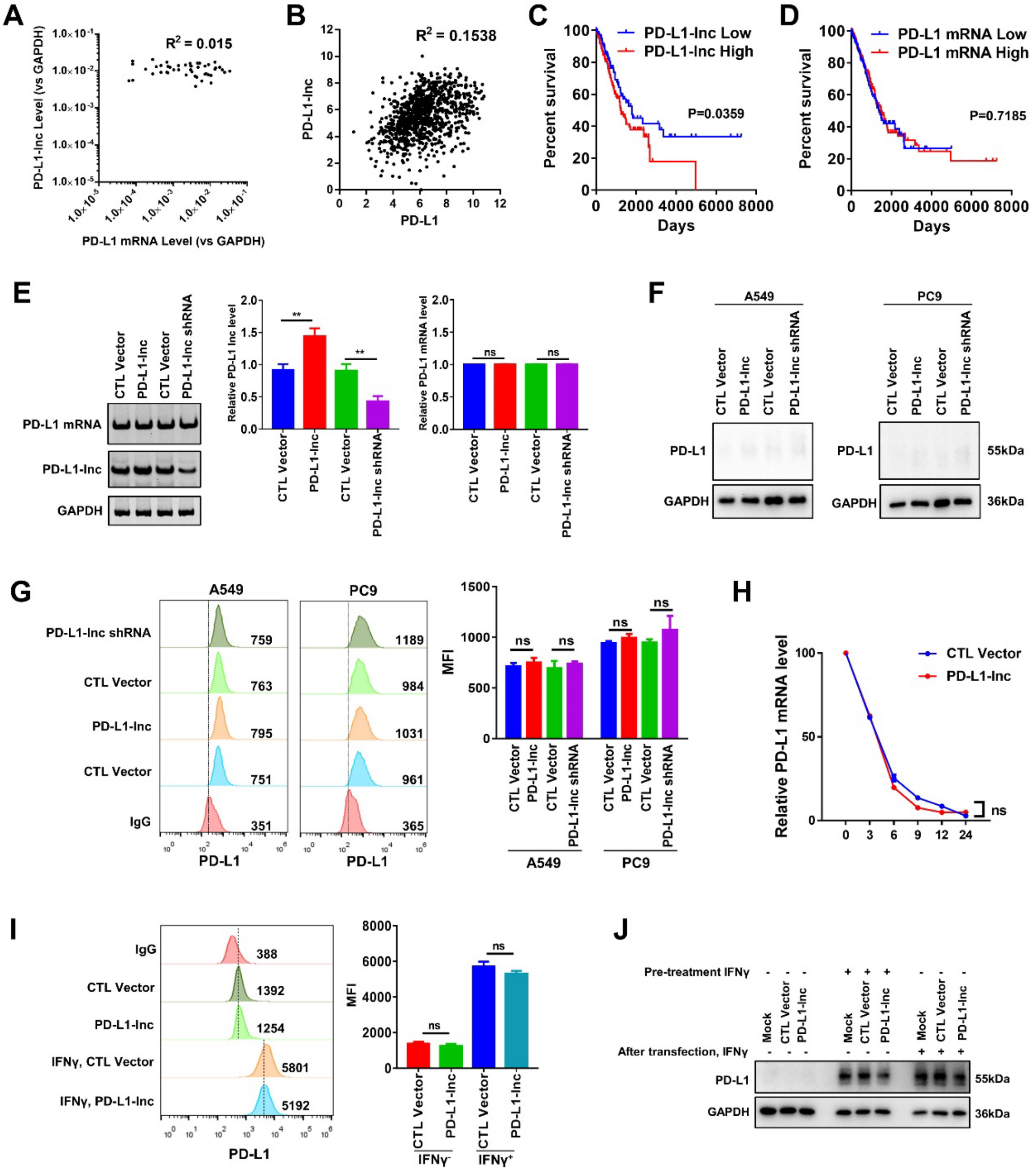


**Figure S7. PD-L1-lnc has no effect on PD-L1 mRNA and protein expression. (A)** Relative expression of the PD-L1-lnc and PD-L1 mRNA in PD-L1 positive and negative lung adenocarcinoma tissues. **(B)** Relative expression of the PD-L1-lnc and PD-L1 mRNA in lung adenocarcinoma data set of TCGA. **(C-D)** Kaplan–Meier survival analysis of lung adenocarcinoma patients stratified by PD-L1-lnc (C) and PD-L1 mRNA (D) expression in TCGA database. **(E)**The expression of PD-L1 lncRNA and mRNA in A549 cells transfected with PD-L1-lnc overexpression vector or PD-L1-lnc shRNA vector. **(F-G)** Western blotting (F) and Flow cytometry (G) analysis of PD-L1 protein level in A549 and PC9 cells transfected with PD-L1-lnc overexpression vector or PD-L1-lnc shRNA vector. **(H)** The half-life of PD-L1 mRNA in A549 cells transfected with PD-L1-lnc overexpression vector or control vector. **(I-J)** Flow cytometry (I) and Western blotting (J) analysis of PD-L1 protein levels in A549 cells transfected with PD-L1-lnc overexpression vector or control vector in the presence or absence of IFNγ. PD-L1-lnc overexpression vector: PD-L1-lnc; control vector: CTL Vector. ***P* < 0.01.

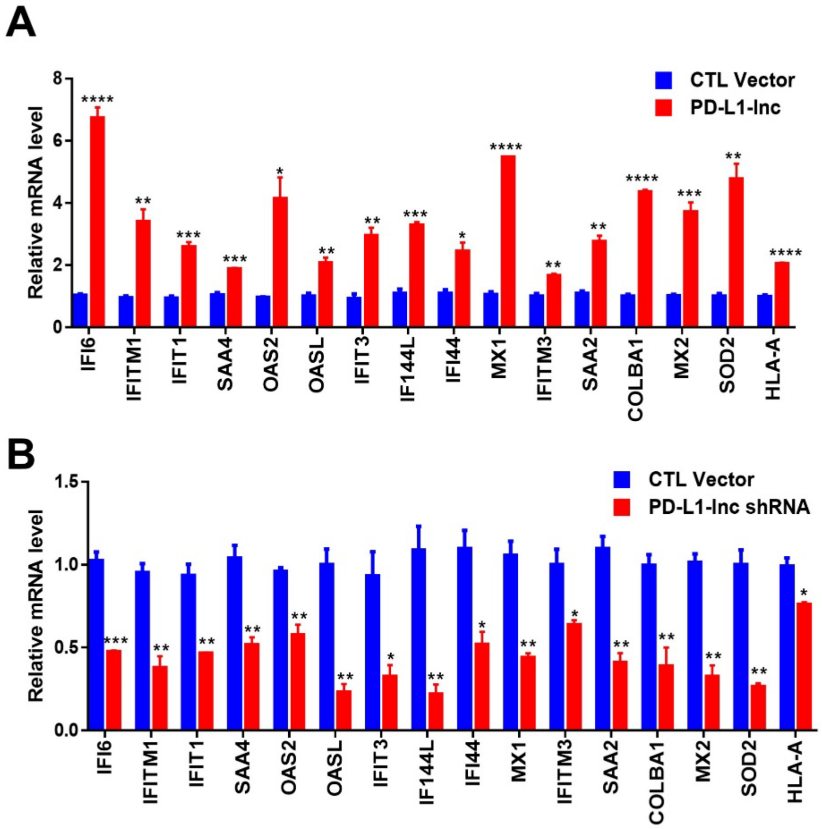


**Figure S8. Expression level of genes within the c-Myc signaling downstream in lung cancer xenograft.** *P < 0.05; **P < 0.01; ***P < 0.001; ****P < 0.0001.

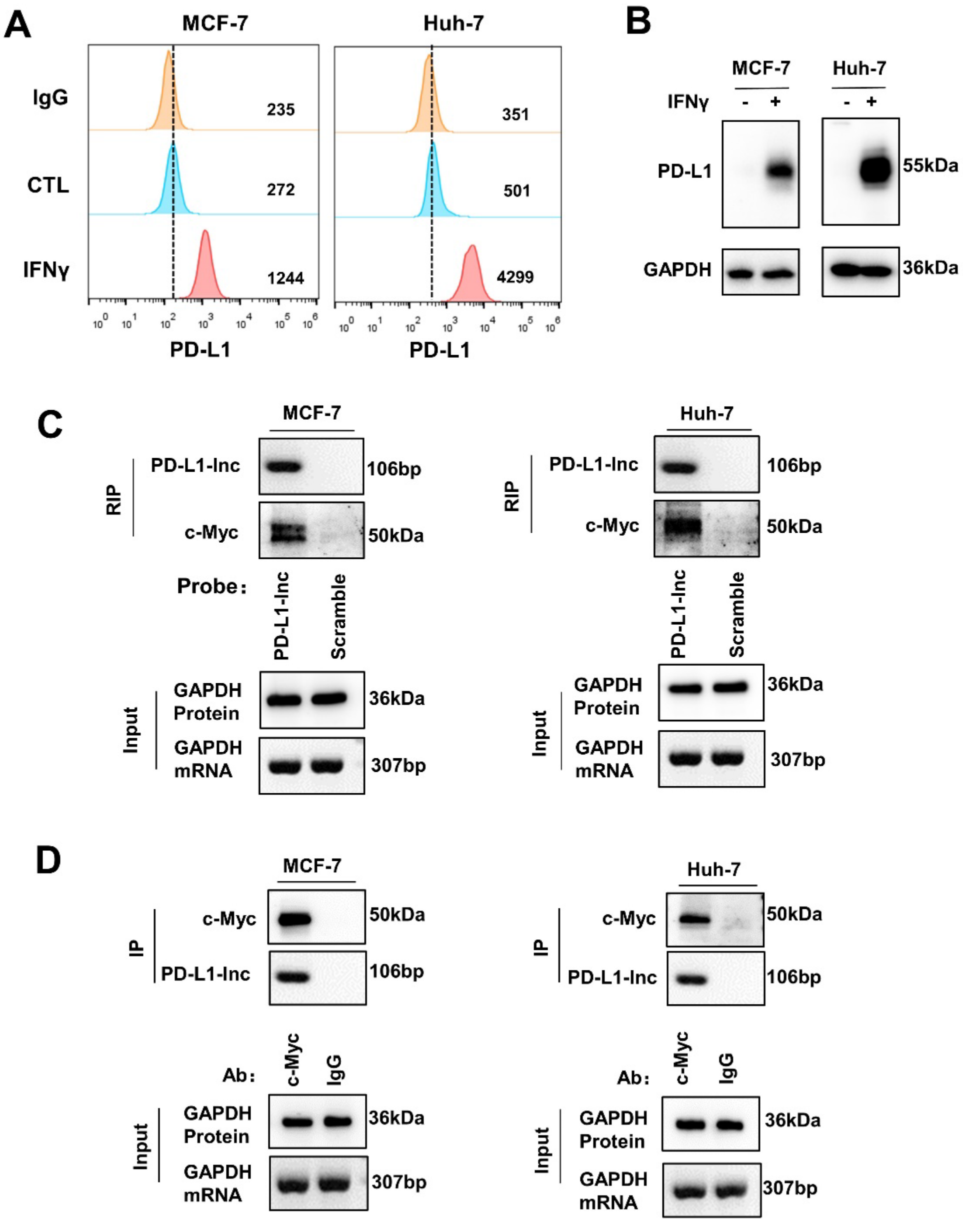


**Figure S9. PD-L1-lnc binds to c-Myc**. **(A-B)** The expression level of PD-L1 protein in MCF-7 and Huh-7 cancer cell line with or without IFNγ stimulation assessed by flow cytometry and Western blotting analysis. **(C)** Immunoprecipitation of PD-L1-lnc in MCF-7 and Huh-7 cells using biotinylated antisense probes against PD-L1-lnc to detect the PD-L1-lnc-associated c-Myc. **(D)** Immunoprecipitation of c-Myc in MCF-7 and Huh-7 cells using anti-c-Myc antibody and Protein A/G beads to detect the c-Myc-associated PD-L1-lnc.

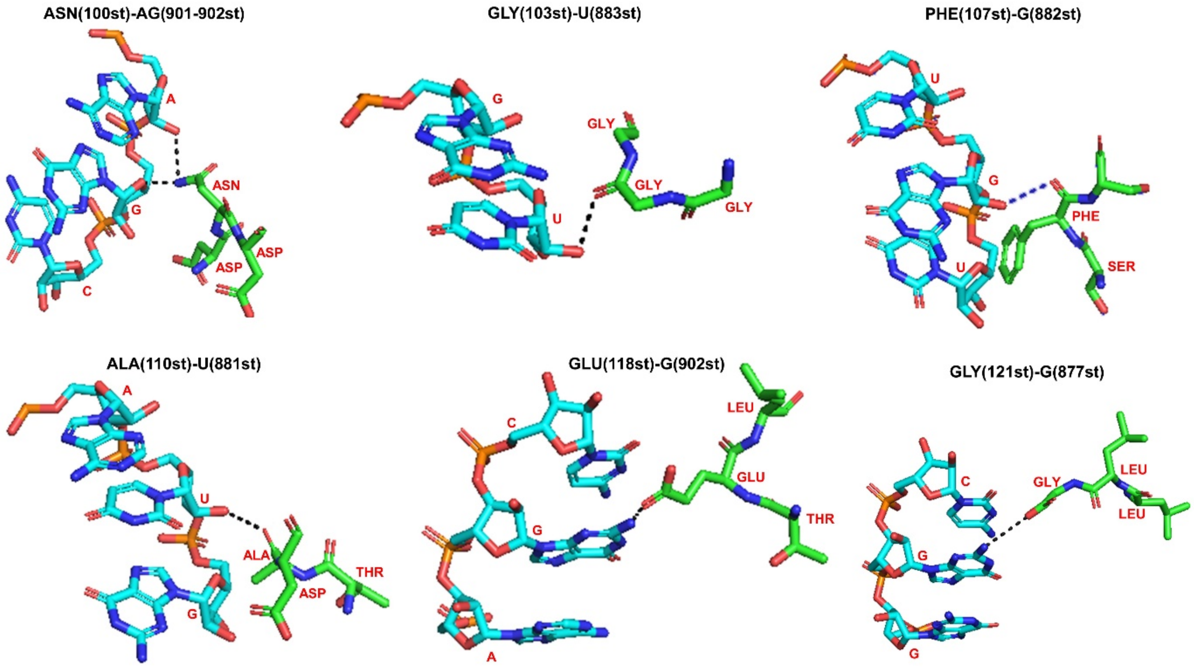


**Figure S10. Graphical representation images of the binding interface of the docking models of PD-L1-lnc with c-Myc by NPDock.** Black dashes represent hydrogen bond.

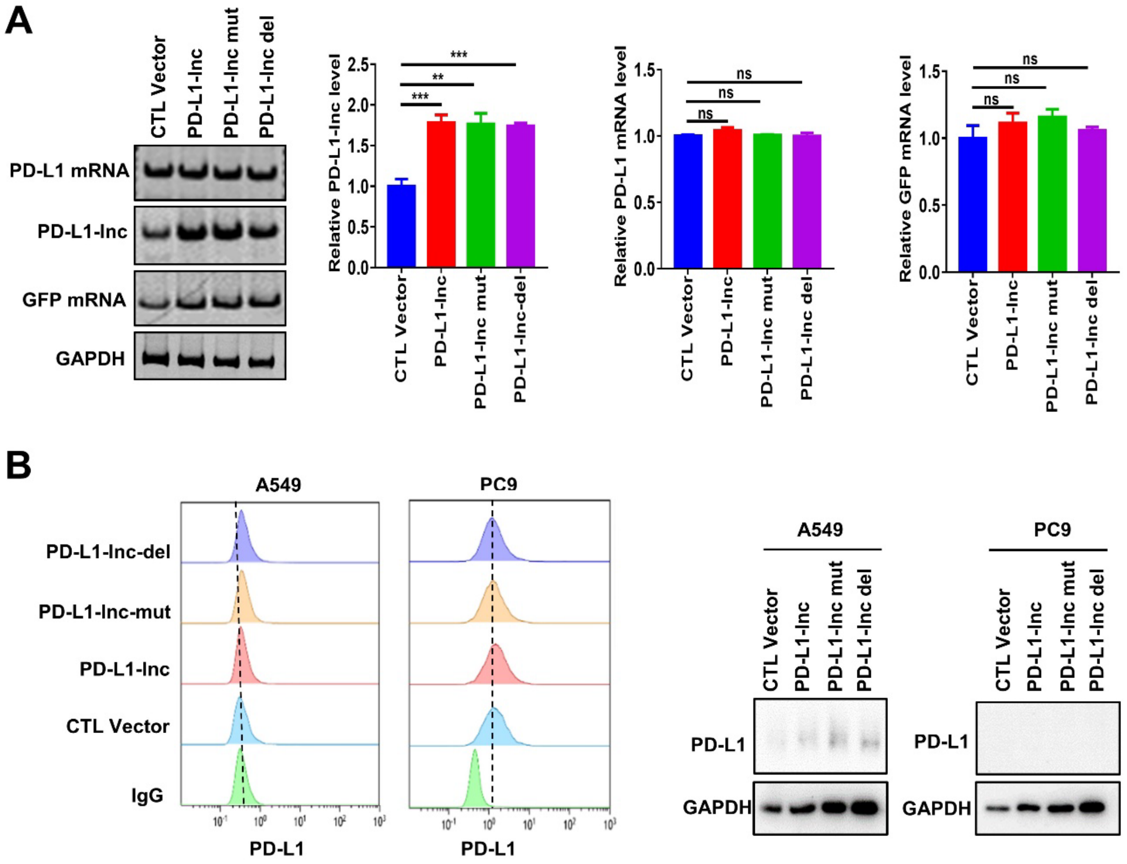


**Figure S11. The expression of PD-L1 lncRNA and mRNA in A549 cells transfected with the mutant vector and wild type vector. (A)** Agarose gel analysis of the expression of PD-L1 lncRNA and mRNA in A549 cells transfected with the mutant vectors and wild type vector. **(B)** Flow cytometry (left) and western blotting (right) analysis of the expression of PD-L1 protein in A549 cells transfected with the mutant or wild type PD-L1 vector. PD-L1-lnc overexpression vector: PD-L1-lnc; control vector: CTL Vector. **$P < 0.01$; ***$P < 0.001$.

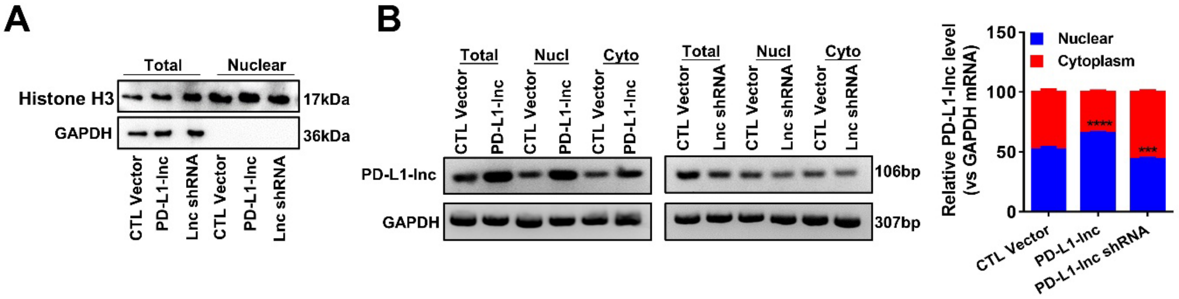


**Figure S12. Cell fraction assay. (A)** Identify the purity of nuclear isolation by Western blotting. **(B)** Cellular distribution of PD-L1-lnc in A549 cells transfected with PD-L1-lnc-expressing vector (PD-L1-lnc), PD-L1-lnc shRNA-expressing vector (PD-L1-lnc shRNA) or control vector. Left: representative image; Right: quantitative analysis. ***$P < 0.001$; ****$P < 0.0001$.

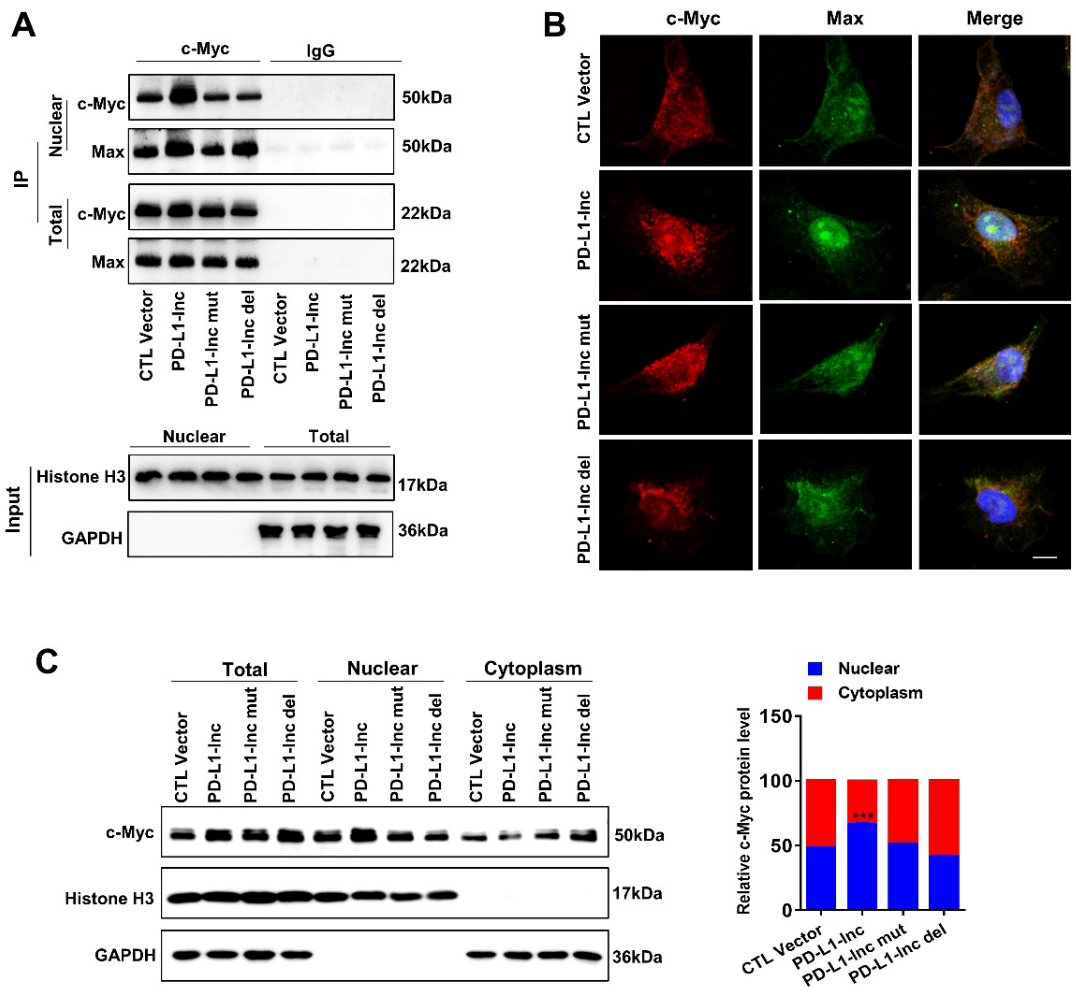


**Figure S13. Binding of PD-L1-lnc to c-Myc enhances the association of c-Myc with MAX and promotes the entry of c-Myc into nucleus. (A)** Pulldown of PD-L1-lnc in A549 cells transfected with WT, PD-L1-lnc mutant, or PD-L1-lnc del vector using biotinylated antisense probes against GFP mRNA to detect the binding of PD-L1-lnc with c-Myc. **(B)** Binding of PD-L1-lnc with c-Myc and Max in A549 cells transfected with WT, PD-L1-lnc mutant or PD-L1-lnc del vector assessed by Immunofluorescence analysis. Scale bar, 10μm. **(C)** Western blot analysis of c-Myc distribution in A549 cells transfected with WT, PD-L1-lnc mutant or PD-L1-lnc del vector. Note that PD-L1-lnc overexpression markedly increases the distribution of c-Myc in A549 cell nuclear fraction, while the PD-L1-lnc mutant and PD-L1-lnc del vector had no effect on the distribution of c-Myc.

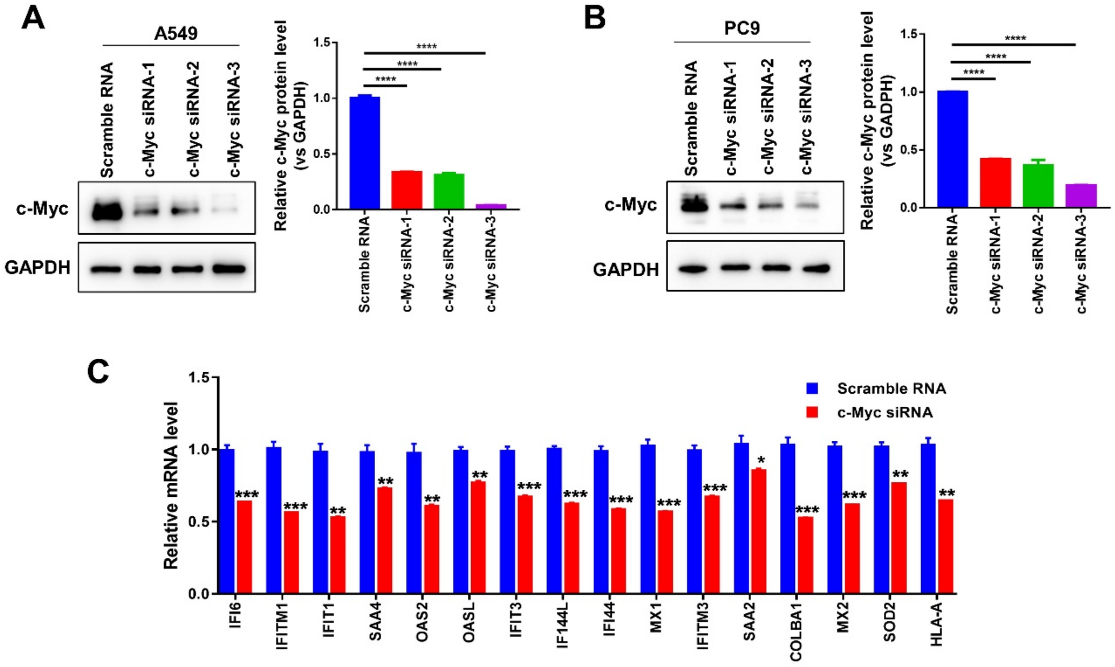


**Figure S14. The influence of c-Myc siRNA on gene expression in lung cancer A549 and PC9 cells. (A-B)** The efficiency of c-Myc siRNA to knock down c-Myc expression in A549 (A) and PC9 (B) cells assessed by Western blotting. **(C)** Expression level of genes associated with c-Myc signal downstream in A549 cells with or without c-Myc siRNA treatment. *$P$ < 0.05; **$P$ < 0.01; ***$P$ < 0.001; ****$P$ < 0.0001.

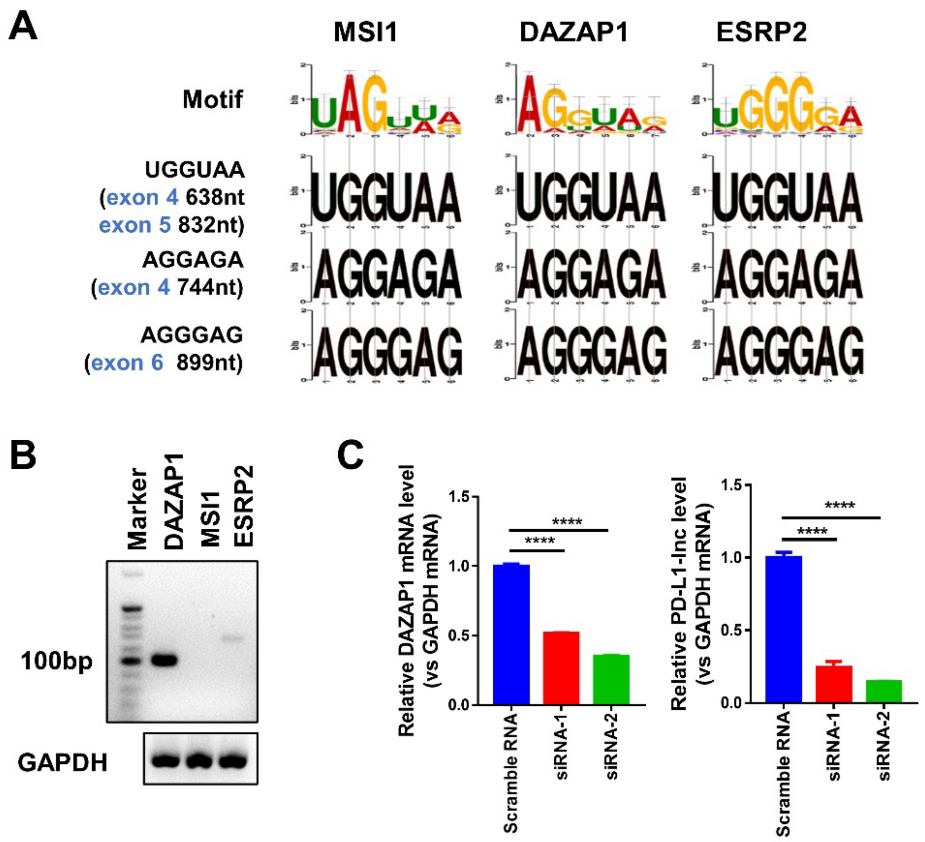


**Figure S15. The effect of DAZAP1 on the generation of PD-L1-lnc. (A)** The motif of MSI1, DAZAP1 and ESRP2 and alternative splicing site of PD-L1. **(B)** The efficiency of DAZAP1 siRNA on DAZAP1 reduction. **(C)** The expression level of DAZAP1 mRNA (left) and PD-L1-lnc (right) in A549 cells transfected with siRNAs to DAZAP1. ****$P < 0.0001$.
