# Supplementary material for: PD-L1 lncRNA splice isoform promotes lung adenocarcinoma progression via enhancing c-Myc activity
Source: Genome Biol. 2021 Apr 13;22:104. doi: 10.1186/s13059-021-02331-0 (PMC8042710; doi:10.1186/s13059-021-02331-0)

Figure 1A

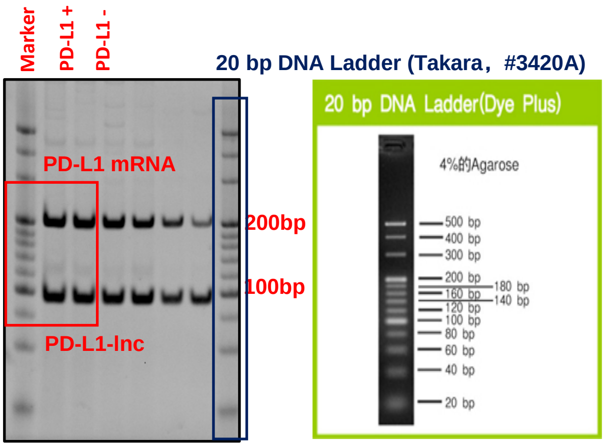

Figure 1D

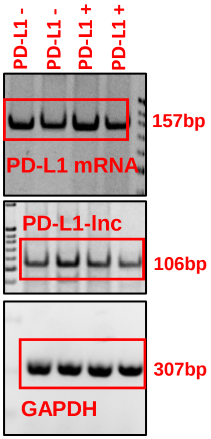

Figure 1B

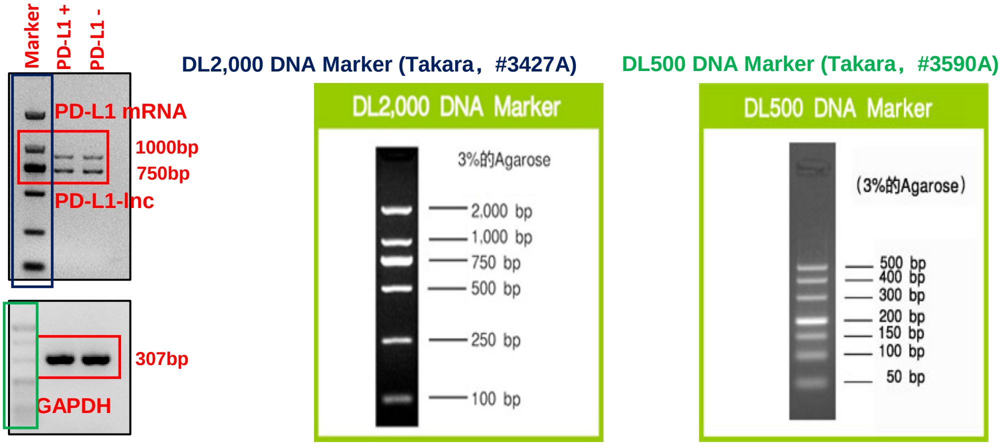

Figure 2A

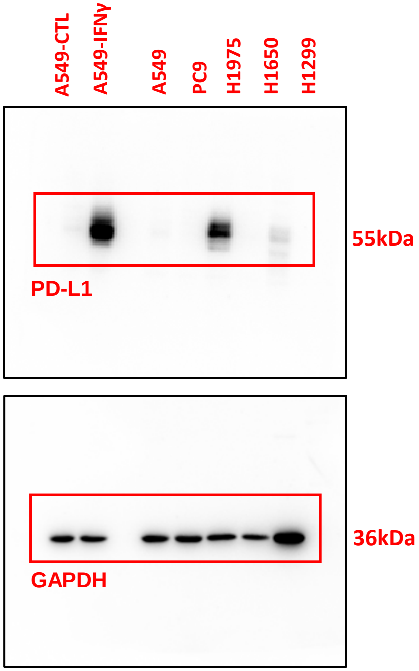

Figure 2C

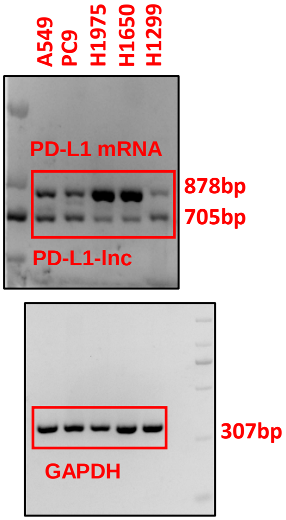

Figure 2E

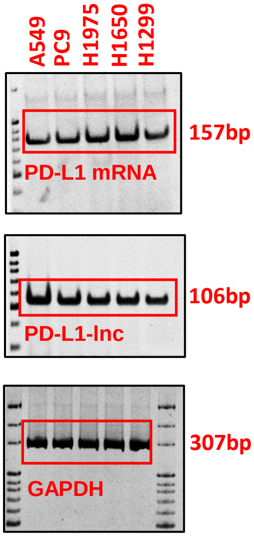

Figure 3B

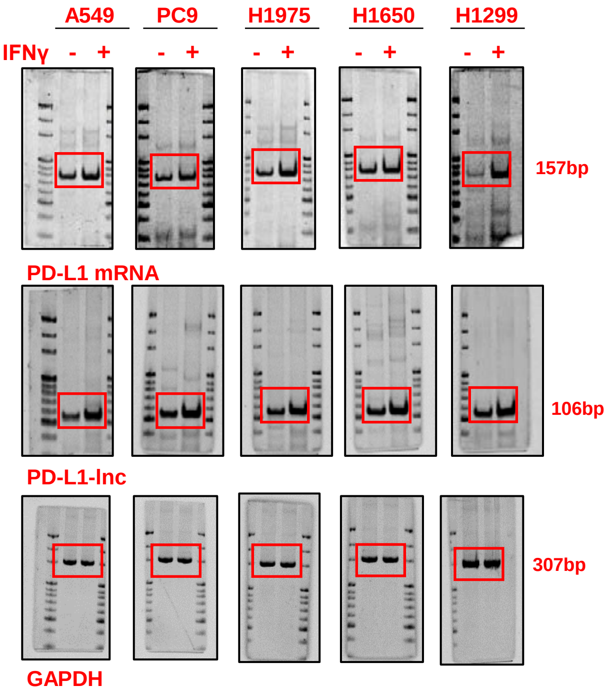

Figure 3C

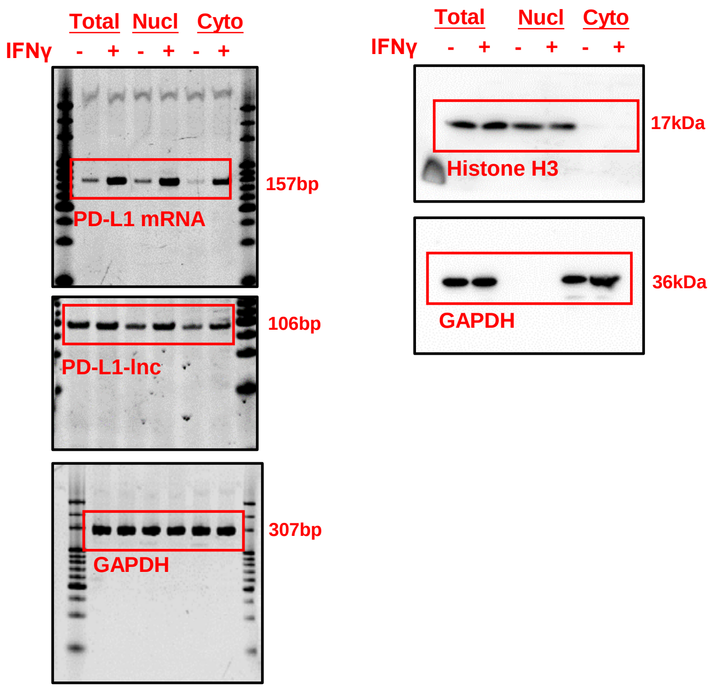

Figure 5C

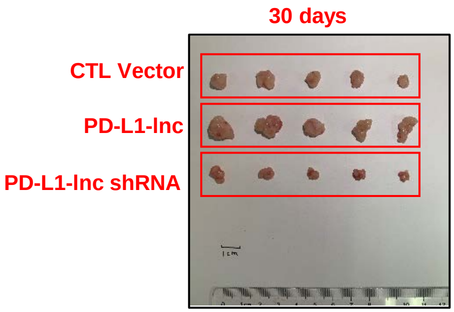

Figure 5E

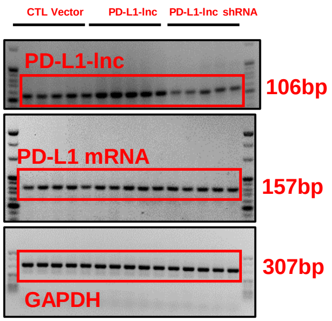

Figure 6D

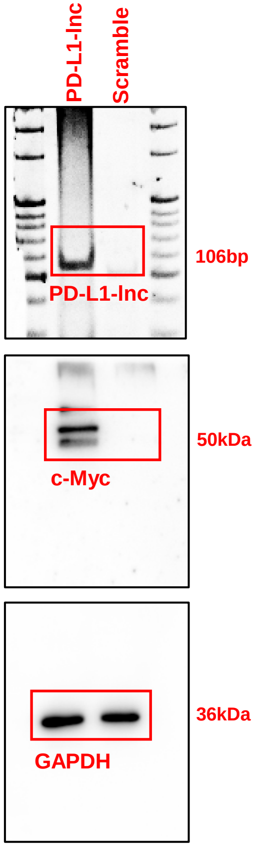

Figure 6E

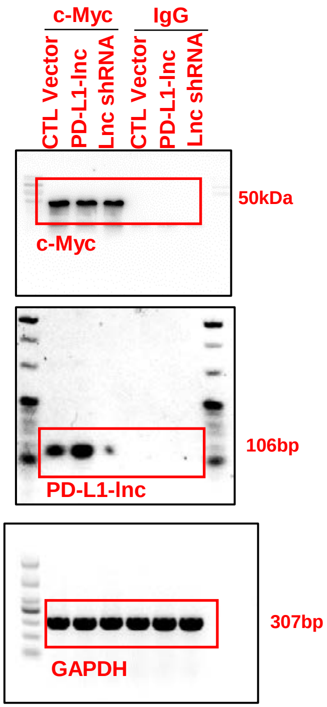

Figure 7C

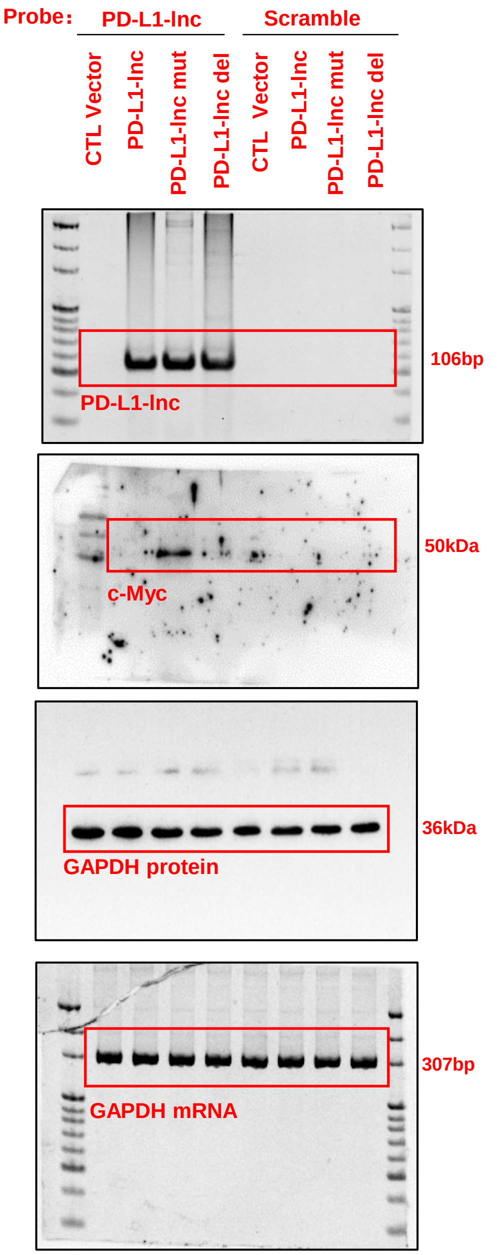

Figure 7D

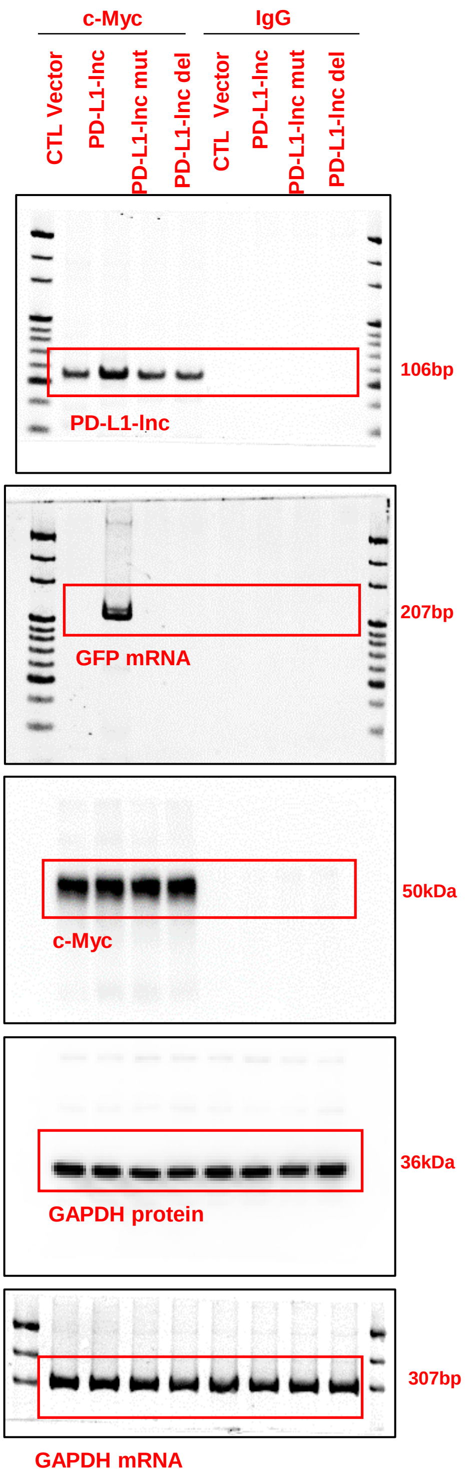

Figure 7E

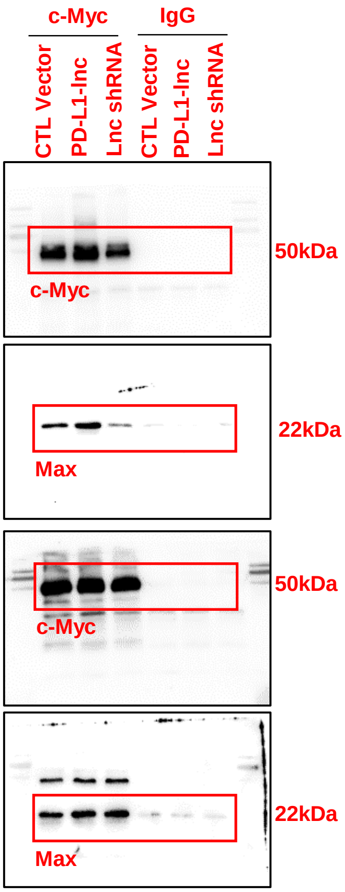

Figure 7G

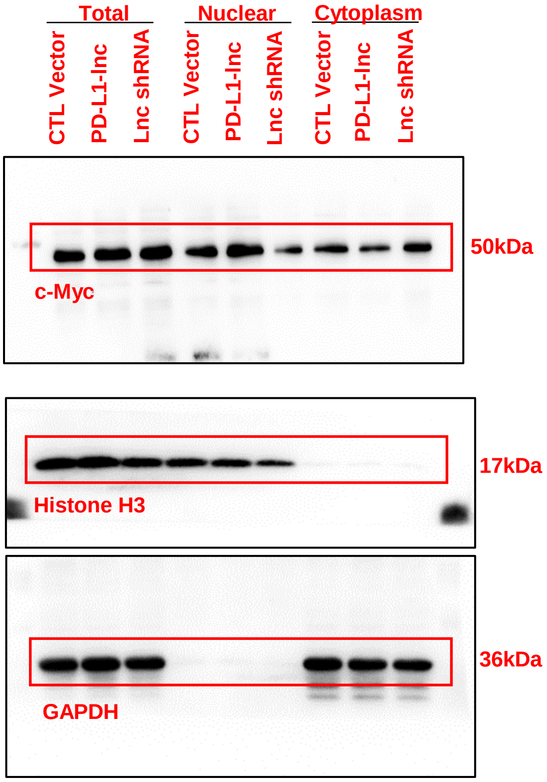

Fig.S4C

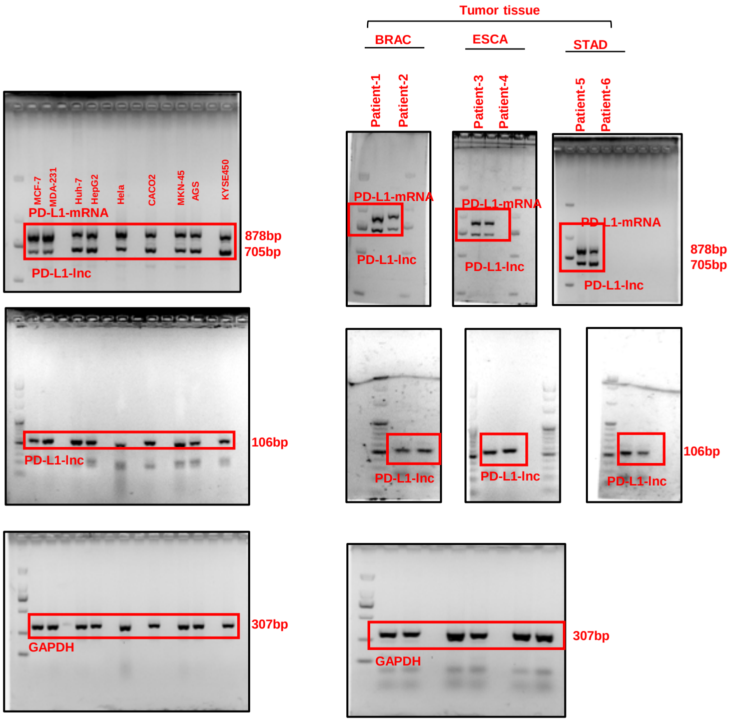
Tumor tissue
BRAC
ESCA
STAD
Patient-1
Patient-2
Patient-3
Patient-4
Patient-5
Patient-6
MCF-7
MDA-231
Huh-7
HepG2
Hela
CACO2
MKN-45
AGS
KYSE450
PD-L1-mRNA
PD-L1-lnc
878bp
705bp
106bp
GAPDH
307bp

Fig.S5A

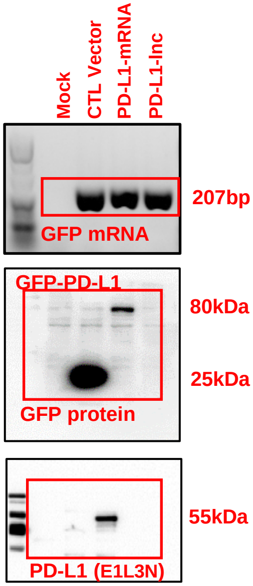

Fig.S5C

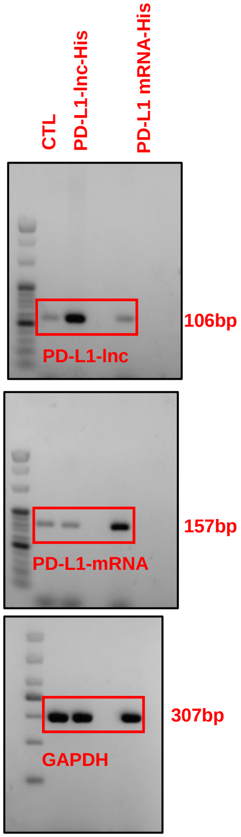

Fig.S5D

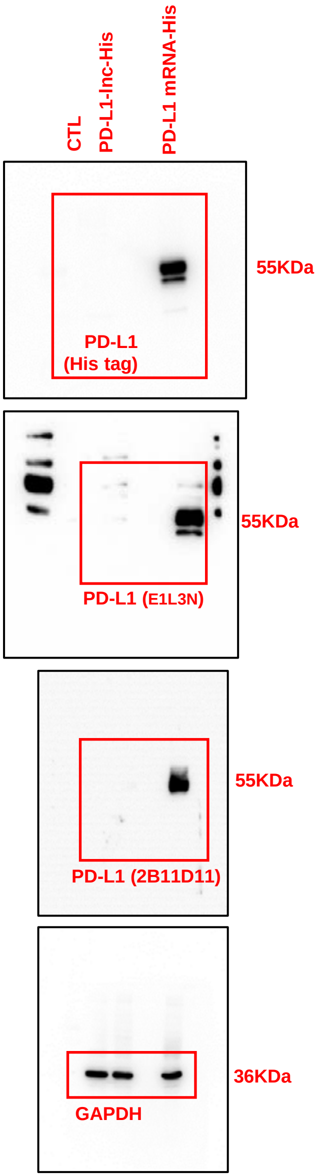

PD-L1 (E1L3N)
Catalog Number:#13684 (CST)
Specifi city/Sensitivity: PD-L1 (E1L3N®) XP ® Rabbit mAb recognizes endogenous levels of total PD-L1 protein.
Source/Purifi cation: Monoclonal antibody is produced by immunizing animals with a synthetic peptide corresponding to residues near the carboxy terminus of human PD-L1 protein.

PD-L1 (2B11D11)
Catalog Number:66248-1-Ig (Proteintech)
CloneNo.:
2B11D11
Immunogen:
PD-L1/CD274 fusion protein Ag12443

Fig. S6A

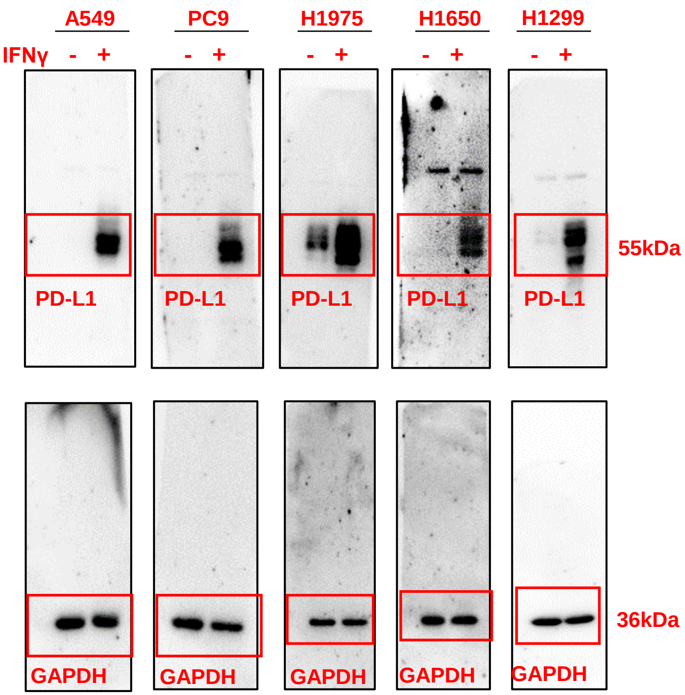
A549
PC9
H1975
H1650
H1299
IFNγ - + - + - + - + - +
PD-L1
PD-L1
PD-L1
PD-L1
PD-L1
55kDa
GAPDH
GAPDH
GAPDH
GAPDH
GAPDH
36kDa

Fig. S7E

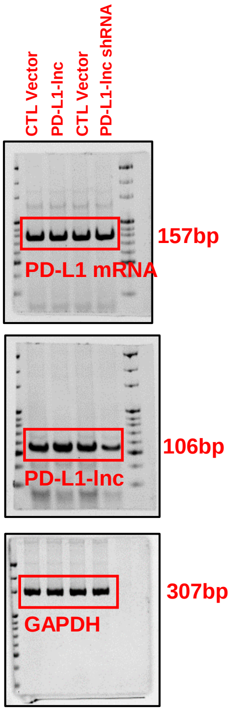

Fig. S7F

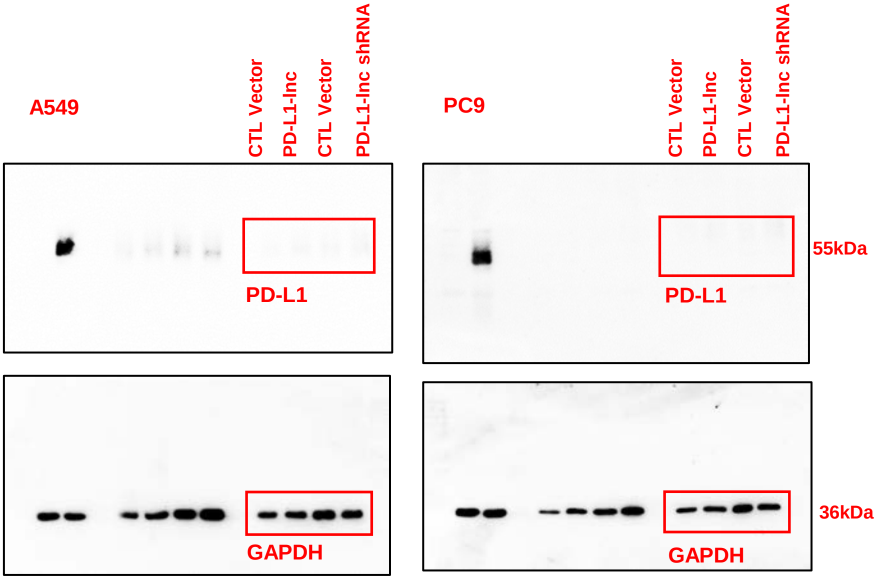

Fig. S7J

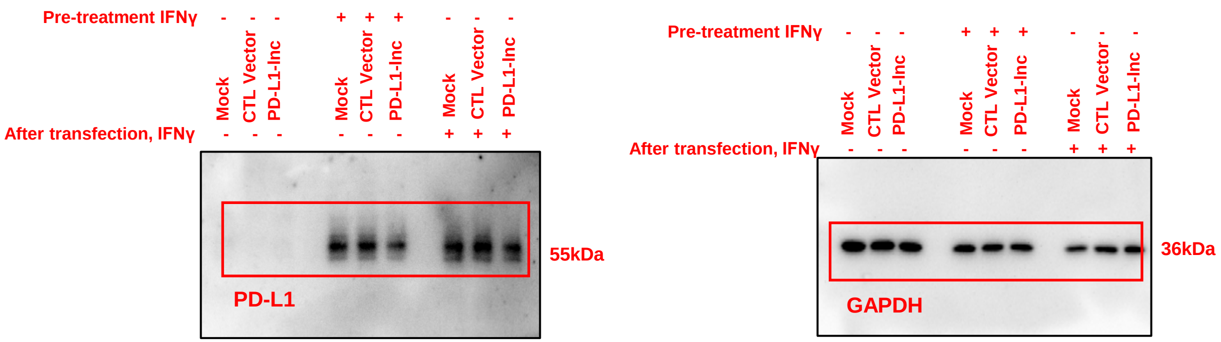

Fig. S9B

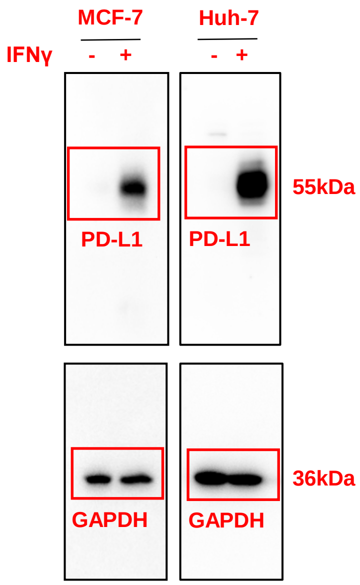

Fig. S9C

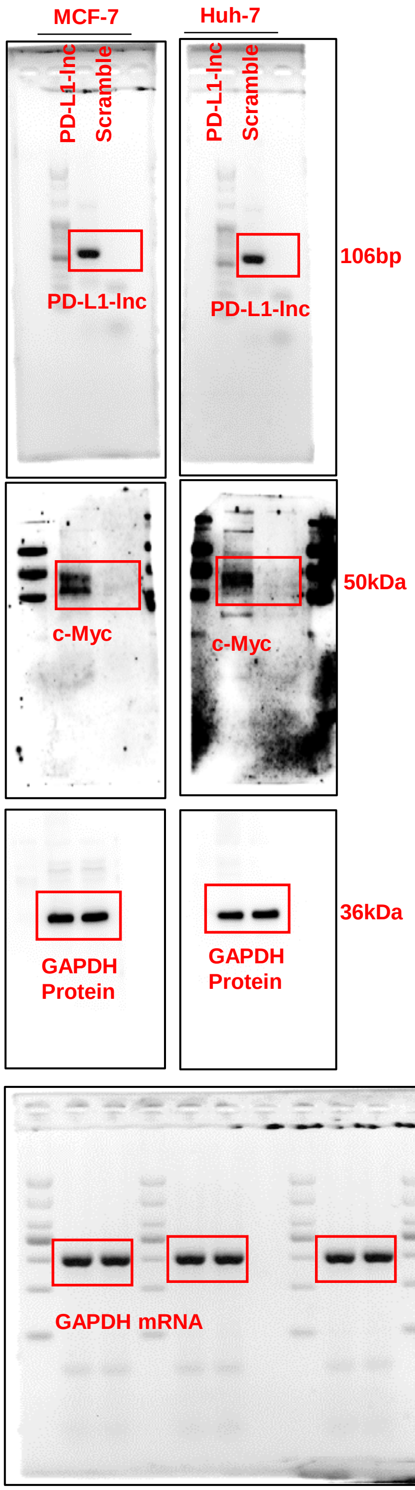

Fig. S9D

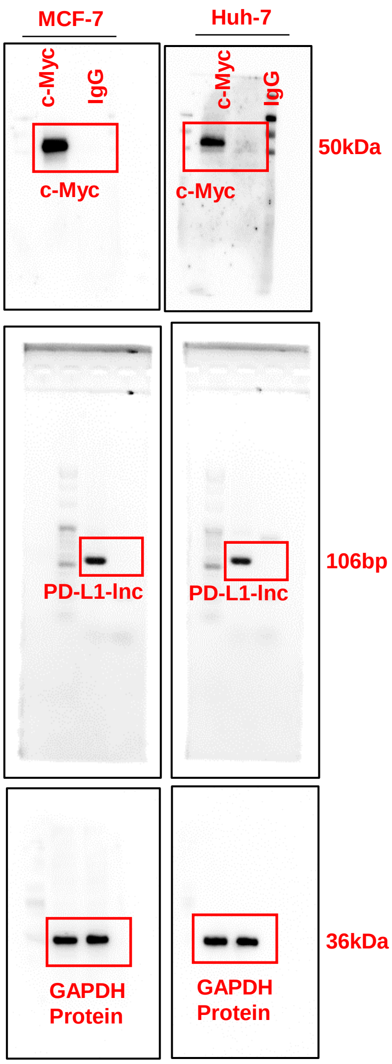

Fig. S11A

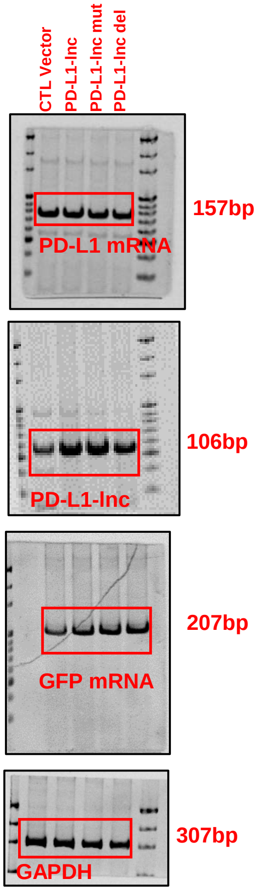

Fig. S11B

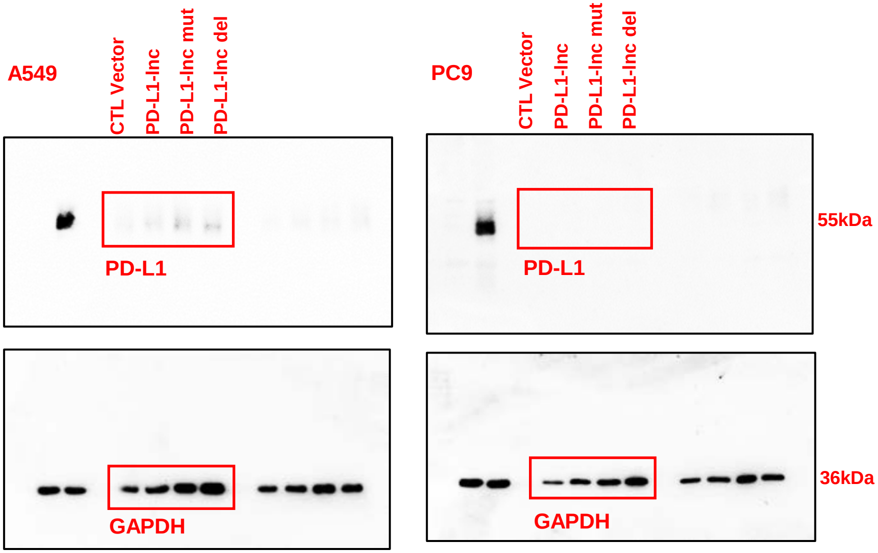

Figure S12A

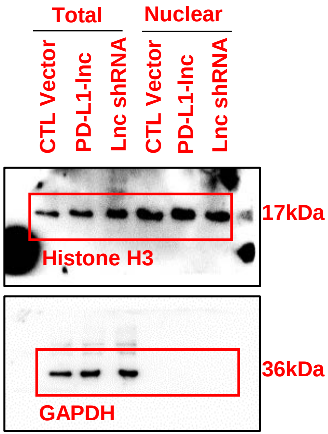

Figure S12B

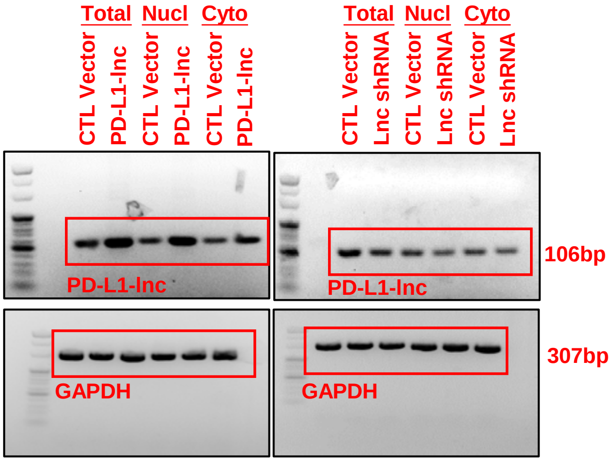

Fig.S13A

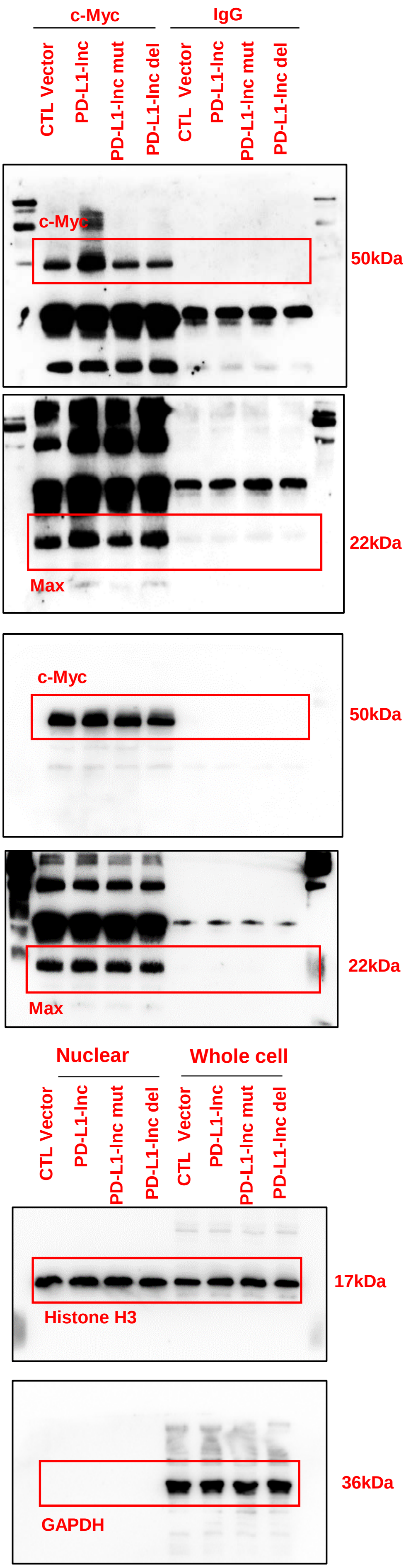

Fig.S13C

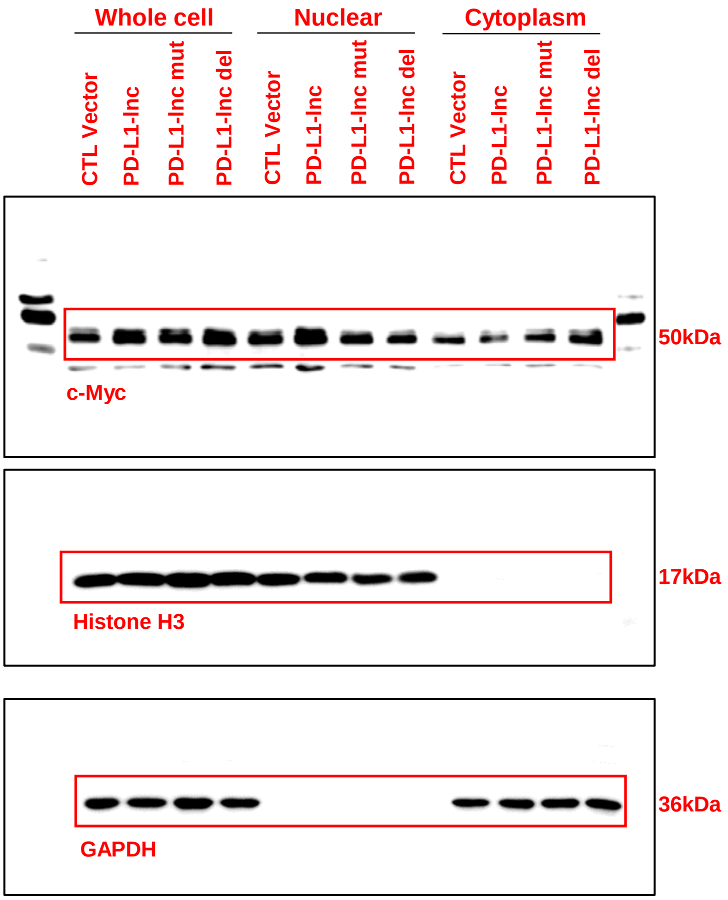

Fig. S14A

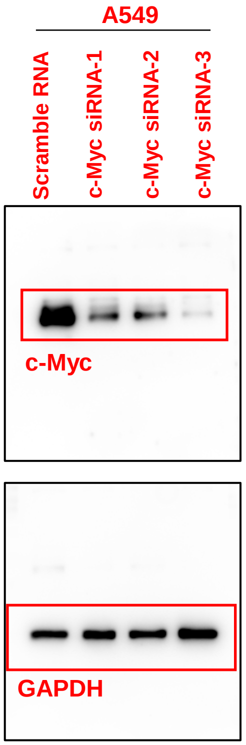

Fig. S14B

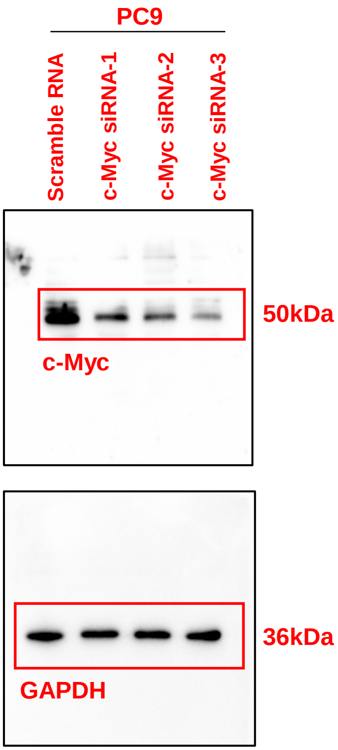

Fig. S15B

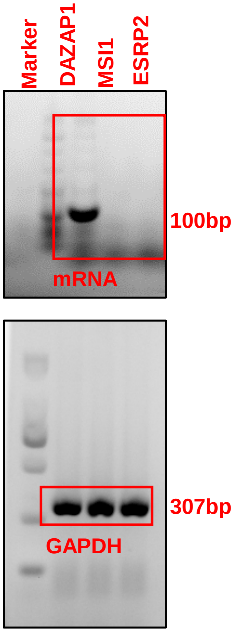

Supplement: Supplementary file 3 — Additional file 3. Source Data for blots. Uncropped Western blots and Agarose gels containing the entire ladder. [file 13059_2021_2331_MOESM3_ESM.pdf]
